# An Engineered Palette of Metal Ion Quenchable Fluorescent Proteins

**DOI:** 10.1371/journal.pone.0095808

**Published:** 2014-04-21

**Authors:** Xiaozhen Yu, Marie-Paule Strub, Travis J. Barnard, Nicholas Noinaj, Grzegorz Piszczek, Susan K. Buchanan, Justin W. Taraska

**Affiliations:** 1 Laboratory of Molecular Biophysics, National Heart Lung and Blood Institute, National Institutes of Health, Bethesda, Maryland, United States of America; 2 Laboratory of Molecular Biology, National Institute Diabetes and Digestive and Kidney Diseases, National Institutes of Health, Bethesda, Maryland, United States of America; 3 Laboratory of Biochemistry, National Heart Lung and Blood Institute, National Institutes of Health, Bethesda, Maryland, United States of America; Cardiff University, United Kingdom

## Abstract

Many fluorescent proteins have been created to act as genetically encoded biosensors. With these sensors, changes in fluorescence report on chemical states in living cells. Transition metal ions such as copper, nickel, and zinc are crucial in many physiological and pathophysiological pathways. Here, we engineered a spectral series of optimized transition metal ion-binding fluorescent proteins that respond to metals with large changes in fluorescence intensity. These proteins can act as metal biosensors or imaging probes whose fluorescence can be tuned by metals. Each protein is uniquely modulated by four different metals (Cu^2+^, Ni^2+^, Co^2+^, and Zn^2+^). Crystallography revealed the geometry and location of metal binding to the engineered sites. When attached to the extracellular terminal of a membrane protein VAMP2, dimeric pairs of the sensors could be used in cells as ratiometric probes for transition metal ions. Thus, these engineered fluorescent proteins act as sensitive transition metal ion-responsive genetically encoded probes that span the visible spectrum.

## Introduction

Since the discovery of green fluorescent protein (GFP) major efforts have been made to identify and create new fluorescent protein (FP) variants with improved photo-physical and photo-chemical properties [1], [2]. There are now many bright stable FPs with unique excitation and emission spectra that span the visible spectrum from blue to far-red. Furthermore, many FPs have been engineered with added functionalities. For example, FPs have been created that respond to cellular conditions such as pH or ions including calcium [3]–[5]. Light-induced photo-activation and photo-switching behaviors in FPs also have been developed and used as optical highlighters for dynamic tracking and super-resolution imaging [6], [7]. Lastly, many FPs have been designed to act as biosensors for enzyme function, cellular conditions, cellular dynamics, and other processes [1], [8]. Many of these FPs with added functions, however, are sub-optimal in color, stability, or brightness compared to the current best evolved FPs. The use of brighter and better-behaved FPs substantially improves the response of these probe systems [9]. Thus, it would be advantageous to rationally design minimal functional modules that could be added to the brightest and best performing FPs to endow these proteins with new behaviors while retaining their superior physical and optical properties.

One feature that has been added to fluorescent proteins is the ability to bind to metal ions [10], [11]. Engineered metal binding sites can increase or decrease the fluorescence of the protein. Thus, metal-induced changes in fluorescence can be used to report the presence of specific metals in a solution or cell. The change in fluorescence induced by metals can occur either by static quenching [12], energy transfer between a colored metal ion and the chromophore [11], or by perturbations to the protein’s structure [10]. In some designs, cross-bridging metal sites have been added to a linker that connects two differently-colored FPs [13]–[15]. When the linker binds to metal the positions of the FPs are changed and the resultant change in Förster resonance energy transfer (FRET) reports metal binding. Additionally, sensors have been created where an FP was used as a scaffold to position an iron-binding protein near the FP choromophore [16].

Here we engineer a palette of bright FPs called “ion-quenchable Fluorescent Proteins” (iq-FPs) whose fluorescence is modulated by the direct binding of transition metal ions to a minimal three histidine metal binding site added to the surface of the protein near the chromophore. These probes are similar to previously designed fluorescent proteins that bind directly to metals [11], [17]. Colored transition metal ions including cobalt, nickel, and copper exhibit concentration dependent and reversible quenching effects when bound to these engineered sites. In one variant, iq-mKate, Zn^2+^ was found to substantially increase the fluorescence of the protein. The concentration and spectral dependence of these effects allows the fluorescence of iq-FPs to be tuned by specific metals. Thus, these probes can act as sensors for metal ions *in vitro* and *in vivo*. Here, we characterize the spectral, structural, and functional properties of this spectral set of engineered metallo-FPs and explore their applications as metal biosensors and metal-modulated imaging probes.

## Results and Discussion

**Figure 1:**
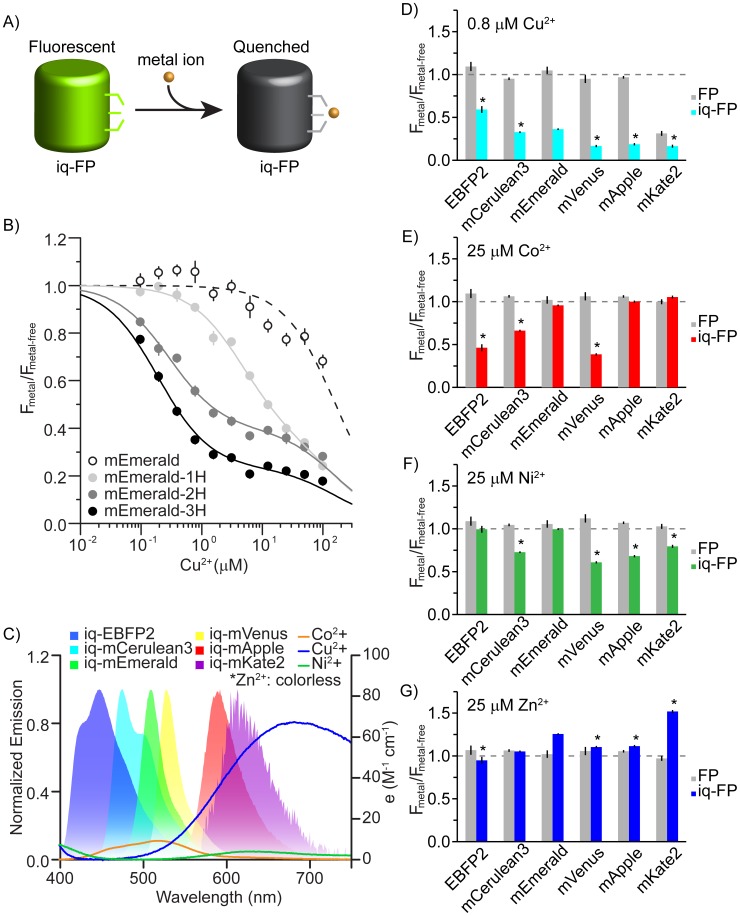
A) Cartoon of experimental design. A metal-binding site is engineered into an FP and fluorescence can be quenched upon the binding of a colored transition metal ion. B) Quenching curves for mEmerald (open circles), mEmerald-1H (H147, light gray), mEmerald-2H (H202 & H204, dark gray), and mEmerald-3H (H147, H202 & H204, black). Spectra are normalized to the fluorescence without metal and the relative fluorescence from each FP is plotted as a function of copper concentration. C) Emission spectra of FPs used in this study and absorbance spectra of three color transition metal ions, Co^2+^, Cu^2+^, and Ni^2+^ (measured with saturating concentrations of EDTA). D) Comparison of fluorescence quenching between FP and iq-FP pairs at 0.8 µM Cu^2+^, E) 25 µM Co^2+^, F) 25 µM Ni^2+^, and G) 25 µM Zn^2+^. Asterisk indicates a significantly different amount of quenching for an iq-FP compared to iq-mEmerald in the presence of metals (p<0.05). All data are average ± S.D. of three independent measurements.

Two surface-exposed histidines separated by three residues on an alpha helix (i and i+4) or one residue in a beta sheet (i and i+2) create a robust transition metal ion binding site in proteins [17]. These minimal motifs have been used to make engineered metal-binding proteins useful for numerous applications including protein purification, functional control, structural mapping, and metal sensing [18], [19]. In some FPs engineered with metal-binding motifs, the binding of metals modulates the fluorescence of the chromophore [10], [11], [13], [20]. Because spectral variants of GFP are similar in structure, we reasoned that minimal metal binding sites could be added to any related FP and used to modulate the fluorescence of these spectrally distinct proteins. Colored metals whose absorbance overlaps the emission of these FPs should quench them by FRET when metals are bound (Fig. 1A).

The strength of a transition metal ion binding site depends on the number, type, and structural positions of the metal-binding residues [18], [19], [21]. To study the effect of added histidines on metal-induced quenching, we first cloned, expressed, and purified four mEmerald constructs: mEmerald (mEmerald), mEmerald-1H (H147), mEmerald-2H (H202 & H204), and mEmerald-3H (H147, H202 & H204). Two of the histidines are spaced one residue apart on strand 10 of the FP (H202 & H204) and the third (H147) was added at the closest position along the neighboring strand (strand 7) to provide a third ligating residue (Fig. S1 and S2). Residue positions were chosen based on a previously designed metal binding green fluorescent protein and the crystal structure of other FP variants [11], [22]–[24]. Figure 1B shows that mEmerald was quenched only at high Cu^2+^ concentrations (>100 µM) (Fig. S3). mEmerald-1H exhibited an added low-affinity (1–10 µM) quenching component. This is similar to data from fluorescently-labeled single histidine metal-binding peptides [21]. Quenching in this mutant was likely the result of weak copper binding to the single added H147 residue. The mEmerald-2H showed stronger quenching behavior with a K_d_ of 0.3 µM. The mEmerald-3H mutant showed the highest affinity (0.2 µM) and greatest quenching effect (80% decreases in fluorescence). These data show that the tri-histidine mutant (which we call iq-mEmerald) provides the most robust and sensitive metal binding site which modulates the fluorescence of mEmerald. The specific K_d_ of the engineered 3H site was determined with a two-site binding model used in previous tmFRET experiments (Fig. S3) [17], [21], [25]. The first site accounts for the low affinity metal quenching seen in the FP controls and the second site accounts for the specific engineered site. No difference in the pH dependence of fluorescence was observed between the mEmerald and the iq-mEmerald (Fig. S4). Also, the presence of physiological concentrations of Ca^2+^ (1 mM) and Mg^2+^ (10 mM) did not change the quenching behavior of iq-mEmerald by copper ions (Fig. S5). These data show that the tri-histidine motif specifically binds soft ligands such as Cu^2+^, Co^2+^, Ni^2+^, and Zn^2+^, but not other divalent ions including Ca^2+^ and Mg^2+^.

Figure 1C shows the emission spectra of the six FP variants we used in this study: EBFP2 [26], mCerulean3 [27], mEmerald [28], mVenus [29], mApple [30], and mKate2 [31], [32]. Each is relatively bright, well folded and monomeric, and their emission spectra overlap the absorbance spectrum of colored transition metal ion FRET acceptors including copper, cobalt, and nickel. For each FP variant we made surface-exposed tri-histidine mutants at similar positions to the iq-mEmerald construct. Aside from the mutant based on mKate2, the excitation, emission, quantum yield, and relative brightness values of these engineered proteins were similar to their parent protein counterparts (Fig. 1C, Fig. S6 and Table 1). From these spectral characteristics we calculated the distances at which each metal will quench the probes by 50% (the R_0_ FRET value, Table S1) assuming a FRET-based mechanism of quenching. For Cu^2+^ the R_0_ values span between 7 and 20 Å (R_0_: iq-EBFP2, 7.2 Å; iq-mCerulean3, 12.3 Å; iq-mEmerald, 13.1 Å; iq-mVenus, 15.1 Å; iq-mApple, 18.4 Å; iq-mKate2, 20.0 Å). Thus, a substantial but distinctive quenching effect for a metal bound near the chromophore of each of these FPs should be observed.

**Table 1:** The absorption, emission, quantum yield, distinctive coefficient, and relative brightness values of all the FPs and iq-FPs.

	Abs max (nm)	Em max (nm)	QY	ε (cm^−1^ M^−1^)	relative brightness
EBFP2	386	447	0.57	22,300	0.34
iq-EBFP2	386	446	0.63	24,800	0.42
mCerulean3	432	475	0.99	27,300	0.72
iq-mCerulean3	433	474	0.92	27,700	0.68
mEmerald	482	509	0.74	51,200	1.00
iq-mEmerald	488	511	0.67	50,400	0.89
mVenus	516	529	0.64	94,800	1.62
iq-mVenus	516	529	0.63	88,700	1.48
mApple	569	593	0.37	43,900	0.43
iq-mApple	568	593	0.36	34,400	0.33
mKate2	588	629	0.38	37,600	0.38
iq-mKate2	580	632	0.34	11,600	0.11

The detailed measurement protocol is described in Materials and Methods section. Relative brightness (QY * ε) is normalized with respect to mEmerald.

Next we measured quenching with four different metals (Cu^2+^, Co^2+^, Ni^2+^, and Zn^2+^) in all the FPs and iq-FPs (3H) spectral variants. Among these four metals, the first three are colored and have been used as transition metal ion FRET acceptors [21], [25]. Figure 1D–1G show that most FPs without metal binding sites do not respond to moderate metal concentrations. iq-FPs, however, substantially but differentially quench in the presence of each colored metal (as shown by the p value of various iq-FPs in Fig. 1D–1G). The pattern of quenching followed the expected amounts according to the R_0_ calculations for each metal/FP pair (Fig. 1D–1G and Table S1). For example, Cu^2+^ has a shorter R_0_ with EBFP2 (7.2 Å) than with mApple (18.4 Å) and the quenching observed in EBFP2 is less (Fig. 1D). The total quenching, however, in each FP is expected to deviate from the predicted levels due to the unique structure of the chromophores, the unknown location energy transfer occurs from, and the exact position of the metals in the structure. Unlike colored metals, zinc has no appreciable absorbance and thus should not act as a transition metal ion-based FRET acceptor. Zn^2+^ showed no substantial quenching effect in these proteins. Surprisingly, iq-mKate2 substantially (∼1.6 fold) increased its fluorescence in the presence of zinc (Fig. 1G). This could be due to a structural stabilization of the beta barrel of the protein. In summary, each iq-FP has different quenching behaviors with each metal ion and their robust and differential response makes them ideal sensors for metals.

**Figure 2:**
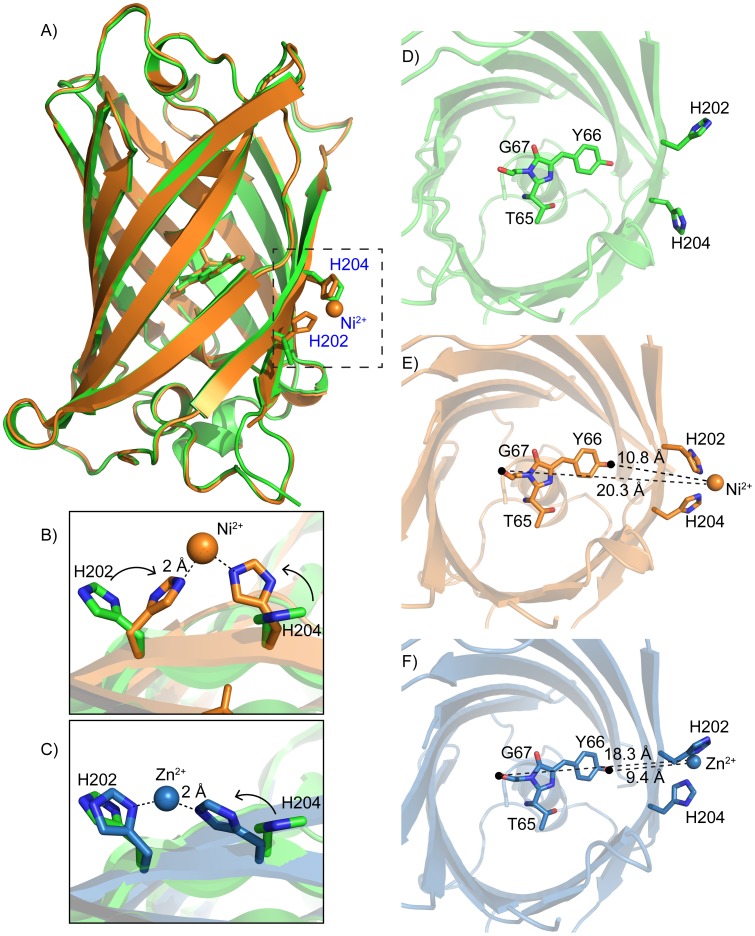
A) Crystal structures of an apo iq-mEmerald (green) and a nickel-bound iq-mEmerald (orange). Two histidines (H202 & H204) that are making contact with the nickel ion are indicated. B) Comparison of the metal-binding site between apo (green) and nickel-bound (orange) structures. C) Comparison of the metal-binding site between apo (green) and zinc-bound (blue) structures. D–F) Top view of iq-mEmerald (from top to bottom: apo (green), nickel-bound (orange) and zinc-bound (blue). The distance from the nickel ion to the mEmerald fluorophore is measured between 10.8 and 20.3 Å. The corresponding distance in the zinc-bound structure is between 9.4 and 18.3 Å.

To understand the structural basis for metal binding we determined the crystal structures of one of our iq-FPs with and without metal ions. We crystallized iq-FP (iq-mEmerald) under three conditions: 1) an apo (PDB: 4KW4), 2) a zinc-bound (4KW9), and 3) a nickel-bound (4KW8) at 1.75 Å, 1.80 Å, and 2.45 Å, respectively (Fig. 2). The apo and zinc-bound iq-mEmerald were solved as a tetragonal P 4_1_ 2_1_ 2 space group. The nickel-bound structure was an orthorhombic P 2_1_ 2_1_ 2_1_ space group (Table S2). Despite these differences, compared to an EGFP structure (PDB: 2Y0G [33]) there was little overall differences in the structures (RMSD = 0.17 Å, 0.25 Å and 0.26 Å, respectively). Likewise, the SYG_65–67_ chromophores were identical (RMSDs of 0.11 Å, 0.18 Å and 0.19 Å in comparison to EGFP). Thus, the tri-histidine site (with and without metals) induces no substantial changes to the overall secondary structure of the FP or its chromophore (Fig. 2A).

In the nickel-bound structure, nickel is coordinated by the two engineered histidines (H202 & H204) and an aspartate (D117) from a neighboring iq-mEmerald in the crystal lattice (Fig. S7). These three residues form an imperfect tetrahedron with the metal at its center. The D117 metal contact is a crystal-packing artifact because iq-mEmerald is a monomer in solution in the presence or absence of metals as indicated by analytical ultracentrifugation data (Fig. S8). The third engineered histidine (H147) points away from the metal and is blocked from interacting with the ion by the D117 residue from the adjacent FP. The increased affinity in the mEmerald-3H over the mEmerald-2H mutant in our fluorescence data (Fig. 1B), however, indicates that H147 does interact with the metal outside the context of the crystal. The nickel is spaced 2 Å from the un-protonated nitrogens of the histidine imidazoles and 2.5 Å from the oxygen of D117 (Fig. 2B). The metal ion position in the zinc-bound structure is similar (Fig. 2C). In both metal-bound structures the two histidines rotate towards each other to bind to the metal ion compared with the metal-free structure. The metal ions are positioned between 9.4–18.3 Å (zinc) and 10.8–20.3 Å (nickel) to the closest and farthest non-hydrogen atom in the chromophore (Fig. 2D–2F). Aside from the position of the histidines, the only other difference in the structures was the orientation of Y145. This tyrosine rotates 180° away from the chromophore and is surface exposed in the zinc-bound structure compared to the nickel and apo structures. Because there is no substantial fluorescence change upon zinc binding, the functional relevance of this zinc-specific structural feature is unknown.

**Figure 3:**
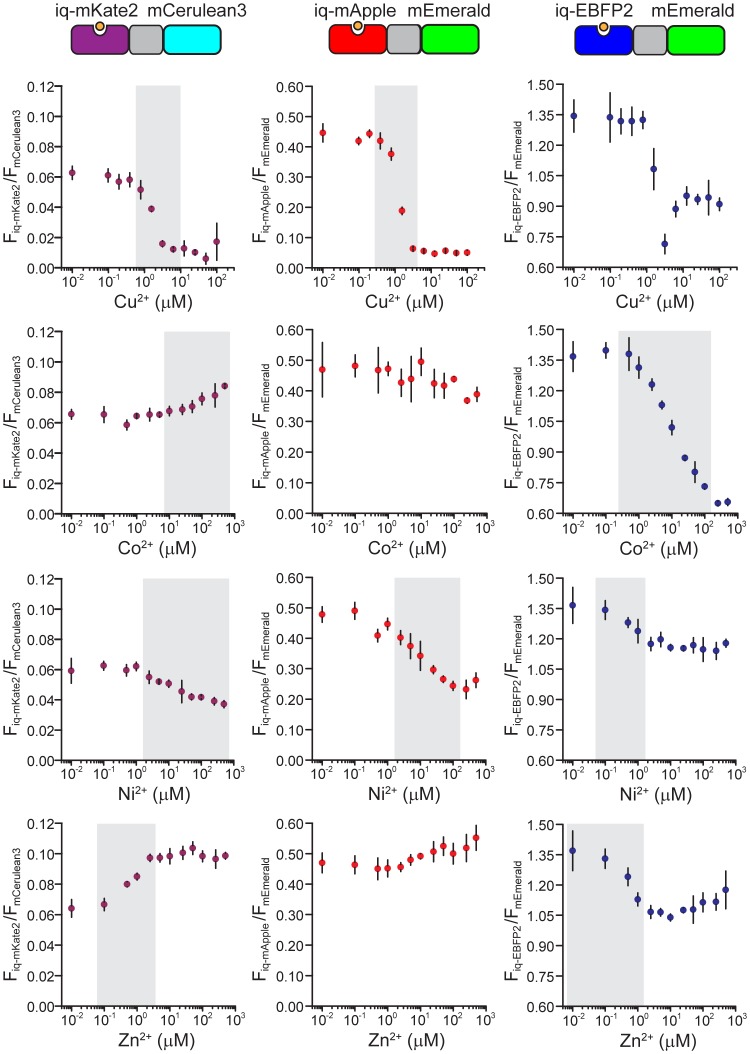
Quenching curves for three ratiometric chimeras, iq-mKate2/mCerulean3, iq-mApple/mEmerald, and iq-EBFP2/mEmerald. Relative fluorescent intensities are calculated by dividing the intensity of iq-FP by the intensity of FP (F_iq-FP_/F_FP_) at each metal concentration. For every chimera, the response to four metal ions, Cu^2+^, Co^2+^, Ni^2+^ and Zn^2+^ are plotted. Gray bars indicate the optimal dynamic range for each chimera and metal ion pair. Although the fluorescence ratios of some chimeras are small, the actual measurement of each component of these chimeras is still well above the background and can be measured with high sensitivity, so the small ratio numbers are not a reflection of the overall sensitivities of these chimeras. All data are average ± S.D.

While single iq-FPs can respond to metal ion concentrations in titration experiments it is difficult to quantitatively measure steady-state levels of ions without a reference marker. To overcome this issue, we designed several iq-FP/FP chimeras to act as ratiometric dimeric sensors for metal ions. These sensors should be able to accurately monitor steady-state levels of metal. To accomplish this we fused a metal-sensitive iq-FP to a metal-insensitive FP with a tetrapeptide GSEF linker. In each pair, the emission spectra of the FP and iq-FP are well separated. Thus, the fluorescence of both could be measured independently. A small amount of energy transfer between iq-FP and FP is possible in these pairs. However, our empirical approach of directly comparing the fluorescent ratios and metal concentrations allows reliable measurement of metals independent of other photophysical effects. Furthermore, in this system because the two FPs are in equal-molar ratio, ratio changes in fluorescence in response to metals should be less sensitive to the expression level of the probes or the excitation levels. Fig. 3 and Fig. S9 shows the response of iq-FP/FP pairs (iq-mKate2/mCerulean3, iq-mApple/mEmerald, and iq-EBFP2/mEmerald). The dynamic range and relative fluorescence ratio change (R_max_/R_min_) in these pairs are summarized in Table S3. We found that for each metal ion, there are two sensors that cover unique dynamic ranges and can be used as sensitive, concentration-dependent metal detectors. Thus, pairs of these probes can be used as new quantitative ratiometric sensors of metal ions over a wide range of concentrations.

**Figure 4:**
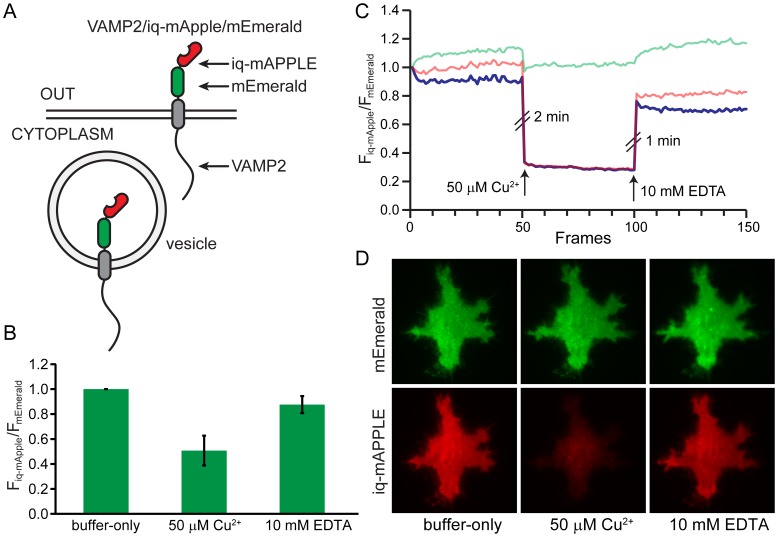
*In vivo* metal quenching experiment with VAMP2/iq-mApple/mEmerald probe. A) A cartoon representation of the construct. The chimeric biosensor iq-mApple/mEmerald is fused to the C-terminus of the membrane protein, VAMP2. A fraction of the sensors are located outside the cell and some are located inside exocytic/endocytic vesicles. B) The fluorescence quenching of VAMP2/iq-mApple/mEmerald by 50 µM Cu^2+^ and its recovery by 10 mM EDTA. Error bars are S.D. C) A representative fluorescence intensity traces with the addition of 50 µM Cu^2+^ and 10 mM EDTA. The raw fluorescent intensities are colored in green and red for two channels and the green/red radio is colored in blue. D) TIRF images from the experiment shown in Fig. 4C.

To test if these probes could be used in living cells we created a fusion of the membrane protein VAMP2 (vesicle-associated membrane protein) with one of our ratiometric probes iq-mApple/mEmerald attached to the extracellular domain of the protein. VAMP traffics through the secretory system and localizes to the plasma membrane, exocytic vesicles, and endocytic vesicles. This fusion is expected to place a fraction of the sensors on the outside of the cell (Fig. 4A). Neuroendocrine PC12 cells expressing the probe showed a strong two-color plasma membrane stain in a total internal reflection fluorescence microscope (TIRF) (Fig. 4D). When 50 µM Cu^2+^ was perfused onto these cells, a robust and specific quenching was observed in the iq-mApple fluorescence while little change was seen in the mEmerald channel. The amount of quenching was 51% ±12% (S.D.) (Fig. 4B), less than that observed with the purified proteins, likely because some of the VAMP was located in intracellular compartments and could not be quenched by extracellular copper. These changes were reversible with the application of EDTA (Fig. 4C and 4D). However, the recovery of fluorescence was not 100% likely because of the combined effects of photobleaching and internalization of surface exposed biosensors. These data show that these probes can be expressed in living cells and respond to metals.

Compared to small organic dyes, genetically encoded probes provide many advantages. They can be tagged to most proteins and cellular compartments and provide a noninvasive method to measure the machinery and signaling pathways within cells. Here, we develop a spectral family of metal-binding iq-FPs that retain the benefits of bright, stable, multi-color FPs, but have added functional responses. These probes can be used as sensors for metal ions including copper, nickel, cobalt, and zinc. Furthermore, the alterations in their fluorescence by metal ions can also be used to chemically tune their fluorescence. These probes are currently some of the brightest and best-behaved metal-binding FPs developed. They additionally span the visible spectrum with emission from the blue to red wavelengths. Furthermore, development of ratiometric paired metal responsive and metal-non responsive FPs in this work allows for the controlled measurement of metals. One limitation of the probes developed here, however, is the fact that they cannot easily discrimination between the color metals Cu^2+^, Ni^2+^, and Co^2+^. Future directed or rational evolution of these probes could be used to improve their metal specificity, binding affinity, or spectral responses. For example, the application of metal specific structural algorithms could be used to design highly specific binding sites within these FP scaffolds [34], [35].

The metal dependent quenching of iq-FPs are similar to other tmFRET-based fluorescence systems [17], [21], [25]. Like those systems, other quenching mechanisms including static quenching and electron transfer are possible. However, because static quenching requires physical contact of the metal and the fluorophore, and electron transfer usually occurs at distances less than 5 Å, we believe these effects to be unlikely because no metals were observed inside the beta-barrel near the chromophore [25]. Furthermore, our data exhibits the typical two components of quenching seen in tmFRET experiments and the close match between our data and the FRET-based models strongly suggests that FRET is the dominant quenching mechanism. The first (high affinity) component likely results from energy transfer between the metal bound to the engineered site and the second (lower affinity) component is due to non-specific solution-phase quenching. This second component is observed even in FPs. From our FRET-specific signal we can estimate the distance between the metals and the chromophore to be an average of 12.2 Å. (Table S1 and Fig. S10). This distance is close to the distance we observed between the nickel ion and the closest atom of the chromophore in the nickel-bound iq-mEmerald crystal structure of 10.8 Å (Fig. 2E). To our knowledge, this is the first direct distance measurement of an intact energy transfer system by both crystallography and fluorescence.

Among the three transition metal ions that we measured, Cu^2+^ has the most dramatic quenching effect (7-fold) at nanomolar concentrations of metal ions. This FP/metal pair is promising as an imaging tool. For example, the probe could be used as an alternative to pH-sensitive GFP (pHluorin) as a reporter for exocytosis and endocytosis [3]. Specifically, adding a solution of copper to a synaptic terminal or cell and measuring the fraction of fluorescence quenched by copper would reveal the amount of iq-FP-tagged membrane proteins released into the plasma membrane during exocytosis. Likewise, two-color imaging could be done on different proteins tagged with the same color probe (an FP and iq-FP). The individual signals could be isolated by taking advantage of their drastically different intensities in metal solutions. In a similar way, iq-FPs could be used to locate specific weak signals from a highly fluorescent non-specific background.

We demonstrate that metal-ion-induced fluorescence changes of iq-FPs could be used as genetically encoded sensors. Metal concentrations are regulated and play important roles in biological systems [36]–[39]. Accumulation of metal ions can cause misfolding or aggregation of proteins that are linked to neurodegenerative diseases, such as Alzheimer’s and Parkinson’s diseases [40]. Thus, measuring the locations and concentrations of these ions is critical. For example, zinc is the second most abundant transition metal in the body and can reach concentrations of 300 µM in the mossy fiber synaptic bouton [41], [42]. Our ratiometric iq-mKate2/mCerulean3 probe offers a dynamic ranges of 0.1 µM –5 µM for zinc with a large emission ratio change (R_max_/R_min_ = ∼1.5) (Table S3). This is similar to other FP-based zinc sensors [10], [20]. Furthermore, its red excitation wavelength is useful in thick scattering tissue like the brain [43]. Like zinc, the concentration of Cu^2+^ can reach 25 µM in blood serum [44] and 30 µM in the synaptic cleft [36]. Our iq-mApple/mEmerald pair can be used to sense copper in these ranges (Table S3).

## Conclusions

Here, we generated a family of iq-FPs and characterized their structural, physical, and optical properties. These probes provide genetically-encoded optical sensors for metal ions that can be used in diverse imaging applications. Their multi-color, multi-ion, and ratiometric nature allows the direct monitoring of metal ions in real time. For both direct metal sensing, and modulated fluorescence applications, these bright well-behaved probes have the potential to act as useful new sensors in cellular and molecular biology.

## Methods

### Plasmids

EBFP2 (pBad-EBFP2, #14891), mVenus (mVenus-N1, #27793), and mApple (Myo1E-pmAppleC1, #27698) constructs were from addgene. mEmerald (mEmerald-C1) and mCerulean3 (pmCerulean3-C1) constructs were generous gifts provided by John Hammer (Laboratory of Cell Biology, National Heart Lung and Blood Institute, National Institutes of Health) and Mark Rizzo (School of Medicine, University of Maryland), respectively. mKate2 (pmKate2-C) construct was purchased from Evrogen. All these FP genes were subcloned into the pMAL-c5x vector (New England Biolabs). The iq-FP constructs were generated by using the QuikChange II XL Site-Directed Mutagenesis Kit (Agilent Technologies). All constructs were sequence confirmed.

### Protein Expression and Purification

All constructs were transformed into BL21(DE3) competent cells (Stratagene). A single colony was suspended in 40 ml LB with ampicillin and incubated overnight at 37°C with shaking at 250 rpm. Two liters of bacteria were grown at 37°C for 4 hours and induced with 1 mM IPTG at OD600 of 0.4 to 0.8. Cultures were grown from 24 to 48 hours at 18°C based on the maturation time of each FP. After centrifugation, cell pellets were re-suspended and lysed with one cOmplete tablet (Roche) and 1 mM PMSF protease inhibitor. The supernatant was cleared by ultracentrifugation at 40,000 rpm and proteins were purified on an amylose column (New England Biolabs). The purity of the samples was confirmed by SDS-PAGE.

### Crystallization, Data Collection and Processing

Crystals of apo iq-mEmerald were obtained by hanging drop vapor diffusion: 0.2 µL of the protein solution (100 mM Tris acid, pH 7.4, 150 mM NaCl, at a concentration of 8 mg/mL) with 0.2 µL of the well solution (50 mM HEPES at pH 8.2, 50 mM MgCl2, 22% PEG4000, 10 mM β-mercaptoethanol) after 48 hours at 21°C. Crystals of nickel-bound iq-mEmerlad were obtained by soaking an apo iq-mEmerald crystal in 10 mM NiCl2 with well solution overnight. Crystals of zinc-bound iq-mEmerlad were obtained by quick-soaking of an apo iq-mEmerald crystal in 1 mM ZnCl2 with well solution for 5 seconds. All crystals were cryoprotected with a mixture of protein buffer and well solution plus 25% glycerol before flash-freezing. Data for the apo and zinc-bound mEmerald structures were collected at wavelengths of 1.00 and 1.28 Å, respectively, at the Southeast Regional Collaborative Access Team (SER-CAT) 22-BM beamline at the Advanced Photon Source, Argonne National Laboratory. Data for the nickel-bound structure were collected at a wavelength of 1.38 Å at the SER-CAT 23-ID beamline (GM/CA). All data were collected at 100 K and were processed with HKL2000 [45]. The structures were solved by molecular replacement using Phaser [46] in PHENIX [47] and the structure of EGFP (PDB ID: 2Y0G) as the search model. All of the structures had one molecule per asymmetric unit. Iterative manual model building and refinement were performed using COOT [48] and PHENIX [47]. Statistics for data collection and refinement are summarized in Table S2.

### Analytical Ultracentrifugation

Experiments were conducted at 20°C using a Beckman Optima XL-I analytical ultracentrifuge (Beckman, Palo Alto, CA) equipped with a four-hole An Ti-60 rotor and cells with 12-mm double-sector Epon centerpieces and sapphire windows. 400 µL of 1 µM iq-mEmerald sample was dialyzed overnight into 2x fluorescence buffer (6 mM HEPES, 260 mM NaCl, pH 7.4) only and 10 µM Cu2+ with 2x fluorescence buffer, respectively. After thermal equilibrium was reached at rest, the rotor was accelerated to 50,000 rpm. Interference and 280-nm absorbance scans were collected continuously until no further sedimentation boundary movement was observed. Data analysis was conducted using the c(s) method in the SEDFIT program [49] and the final plot was done by GUSSI.

### Spectroscopy

All the buffers in this study were treated with chelex-100 column to eliminate trace metal ions in solution. Protein samples were diluted to 0.4, 0.8, 1.2, 1.6 and 2.0 µM in 2x fluorescence buffer and their absorbance spectra were recorded with a Cary 300 Bio UV-Vis spectrophotometer. The absorbance and concentration were fitted into a linear equation and the extinction coefficients (ε) were calculated. Fluorescence measurements were performed in a 96-well plate reader attached to a fluorometer (Fluorolog3-22 and MicroMax 384, Horiba Jobin Yvon). The reference dye sampler kit (Molecular Probes) including Quinine sulfate, Fluorescein, 5-Carboxytetramethylrhodamine, Sulforhodamine 101, and Nile blue perchlorate were used as references in characterizing protein quantum yields (QY). The same concentrations of protein samples and buffer solution were used in measuring quantum yields and emission spectra.

### Steady-State Fluorescence

For metal quenching curves, 100 µL of FP solution (500 nM in 2x fluorescence buffer) was mixed with an equal amount of metal solution in water. Twelve wells of the 96-well plate contained metal solutions ranging from a final metal concentration of 0 to 500 µM. A Narrowed 5-nm emission window was used to speed up data acquisition with a 5-nm slit width was chosen for both excitation and emission. The excitation/emission wavelengths used in this study were: for EBFP2, 371 nm/444–448 nm; mCerulean3, 400 nm/471–475 nm; for mEmerald, 432 nm/506–510 nm; for for mVenus, 452 nm/525–529 nm; for mApple, 517 nm/586–590 nm; for mKate2, 549 nm/614–618 nm.

### FRET Data Analysis

All the fluorescence measurements were blank corrected and done in triplicate. For every FRET pair, a total of 12 metal concentrations were used in fitting the binding curve. A single-site model was used for fitting the FPs to account for nonspecific solution quenching by the metals:
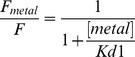
where Fmetal and F are the fluorescence of FPs with and without metal, respectively. Kd1 is the dissociation constant for free metal ions in solution quenching. For iq-FPs, a two-site binding equation was used:



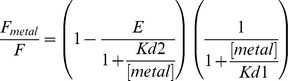
where Kd1 is fixed from the single-site model to account for the nonspecific metal binding. The term on the left represents the FRET effect. Kd2 is the dissociation constant for the engineered tri-histidine metal binding site and E is FRET efficiency. The distance (R) between the chromophore of a FP and the metal ion can be calculated using the Förster equation:



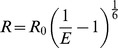
where R_0_ is the Förster distance for a particular FRET pair and its calculation was described previously [50]. Both R and R_0_ were calculated and reported in Table S1. Detailed examples of the data-fitting process were shown in Fig. S9 (for iq-mApple/mEmerald).

### Live Cell Imaging and TIRF Microscopy

PC12-GR5 cell stocks were cultured as described previously [51]. Briefly, cells were plated onto poly (L-lysine)-coated 25 mm glass coverslips. Cells were transfected with 1 µg of plasmid DNA using lipofectamine 2000 (Invitrogen) according to the manufacturer’s instructions. Cells were imaged with an inverted microscope (IX-81; Olympus) configured for TIRF and equipped with a 100x 1.45 NA objective (Olympus) and an image splitter (Dual View, Photometrics) for the simultaneous imaging of red and green fluorescence. Fluorescence was excited by a laser at 488 nm (Melles Griot series 43 ion laser) and 561 nm (Melles Griot LD-561-20A). Lasers were combined and then controlled with an acousto-optic tunable filter (Andor). Optical filters were bright-line full multiband LF405/488/561/635 filters (Semrock). The resulting emission was then divided by the image splitter’s dichroic (565DCXR) and projected side-by-side through 525Q/50 and 605Q/55 emission filters onto the chip of a back-illuminated EMCCD camera (Andor DU 897). Images were acquired using IQ software (Andor). For precise alignment of the red and green images, we imaged 100 nm fluorescent beads (Invitrogen) that fluoresced in both the red and green channels. Regions were aligned by matching the bead locations between the two regions of interest. Pixel size was 167 nm. Frames were acquired in time-lapse recordings with alternating 488 nm and 561 nm excitation with 100 ms exposures given at 2 Hz. For imaging, cells were maintained in imaging buffer (in mM: 130 NaCl, 2.8 KCl, 5 CaCl_2_, 1 MgCl_2_, 10 HEPES, 10 glucose, pH 7.4). Metals and chelators were added by superfusion and complete buffer exchange with individual solutions from a custom perfusion system in the imaging chamber. Experiments were carried out at 28°C.

## Supporting Information

Figure S1 **Sequence alignment of all the FPs used in this study, including mEmerald, mVenus, mCerulean3, EBFP2, mApple, and mKate2.** The sequence of EGFP is also included as a reference. Red boxes and arrows indicate the mutation sites for the tri-histidine metal binding motif. The numbers are arbitrary sequence numbers that are based on the alignment profile.
(TIF)

Figure S2 **A topology diagram of the iq-mEmerald folding pattern.** The β-sheet strands are shown in arrows, α-helices in ribbons, and loops in gray lines. The position of the chromophore is indicated in green and engineered the metal binding histidines are highlighted in red.
(TIF)

Figure S3 **Example data and fit for fluorescence measurements along with the processes to calculate distances.** A and B are the fluorescence emission spectra of mEmerald and iq-mEmerald, respectively. The peak intensities are plotted as respect to copper concentrations into C and D. Then the mEmerald quenching data is fitted with a non-specific single binding site equation (shown as the dotted line in C). The fitting result, K_d_1 of 161 µM, represents the non-specific quenching of the chromophore by metal solution. Then the quenching of iq-mEmerald is fitted with a two binding site equation (shown as the solid line in D). A binding affinity of 0.19 µM and FRET efficiency of 0.77 are obtained from the fitting. Lastly, the distance (10.7 Å) between the chromophore of iq-Emerald and the metal ion is calculated using the Förster equation and reported in Table S1.
(TIF)

Figure S4 **Comparison of the pH dependence of mEmerald (blue) and iq-mEmerald (red).** 500 nM of each protein was incubated with different pH buffer solutions. No difference in the pH dependence of fluorescence was observed in iq-mEmerald.
(TIF)

Figure S5 **Quenching curves for iq-mEmerald by copper ions without (black) or with the presence of physiological concentrations of calcium (1 mM, red) and magnesium (10 mM, green) ions.** Spectra are normalized to the fluorescence without metal and the relative fluorescence from each FP is plotted as a function of copper concentration.
(TIF)

Figure S6 Comparison between the emission spectra of FPs (top) and iq-FPs (bottom) used in this study. The spectra are nearly identical. The absorbance spectra of three color transition metal ions, Co^2+^, Cu^2+^, and Ni^2+^ are plotted for reference.
(TIF)

Figure S7 (Top) Crystal contact interactions between two adjacent iq-mEmerald molecules. (Bottom) Zoom-in of the nickel-bound crystal structure. H202 and H204, along with D117 from the neighboring molecule, made direct connect with the nickel ion. H147 was not able to bind this nickel atom due to this crystal contact.
(TIF)

Figure S8 **Overlay of the sedimentation coefficient distributions for 1 µM iq-mEmerald (black) and 1 µM iq-mEmerald with 10 µM Cu^2+^ (red).** The mass average s values for the monomer peaks in the Emerald c(s) are 4.15S and 4.27S, for the apo- and Cu-bound iq-mEmerald, respectively. The similar sedimentation coefficient distribution of iq-mEmerald indicates that metal ions do not dimerize iq-mEmerald.
(TIF)

Figure S9 **Process used to generate the quenching curves in** **Figure 3** **.** This example is the measurement of a ratiometric chimera construct, iq-mApple/mEmerald (middle panel on the first row of Figure 3). A and B are the normalized emission spectra from the contribution of iq-mApple and mEmerald of the chimear, respectively. The peak fluorescence of both underdifferent copper concentrations are taken and plotted as a fluorescent ratio of F_iq-mApple_/F_mEmerald_ in C and reported in Figure 3. The excitation wavelengthes for this dimeric construct were the same as the single iq-FPs (432 nm for Emerald and 517 nm for mApple in this example).
(TIF)

Figure S10 **Plot of the distances between FRET donor (chromophores of iq-FPs) and acceptors (metal ions) pairs, calculated from FRET measurements.** The dotted lines are the reference distances measured from the nickel-bound iq-mEmerald crystal structure. In this crystal structure, the distance between the nickel ion to the closest and furthest atom on the chromophore are 10.8 and 20.3 Å, respectively. Despite comparing different FP and metal types, most of the FRET calculated distances are within the range that the crystal structure indicated.
(TIF)

Table S1 **The spectrally calculated R_0_ and distances calculated from FRET measurements for each iq-FP/metal pair.**
(PDF)

Table S2 **Data collection and refinement statistics of the iq-mEmerald crystal structures.**
(PDF)

Table S3 **The dynamic range and relative fluorescence ratio change (R_max_/R_min_) in three ratiometric metal sensors, iq-mKate2/mCerulean3, iq-mApple/mEmerald, and iq-EBFP2/mEmerald.**
(PDF)
