# Supplementary figures and images for: An Engineered Palette of Metal Ion Quenchable Fluorescent Proteins
Source: PLoS One. 2014 Apr 21;9(4):e95808. doi: 10.1371/journal.pone.0095808 (PMC3994163; doi:10.1371/journal.pone.0095808)

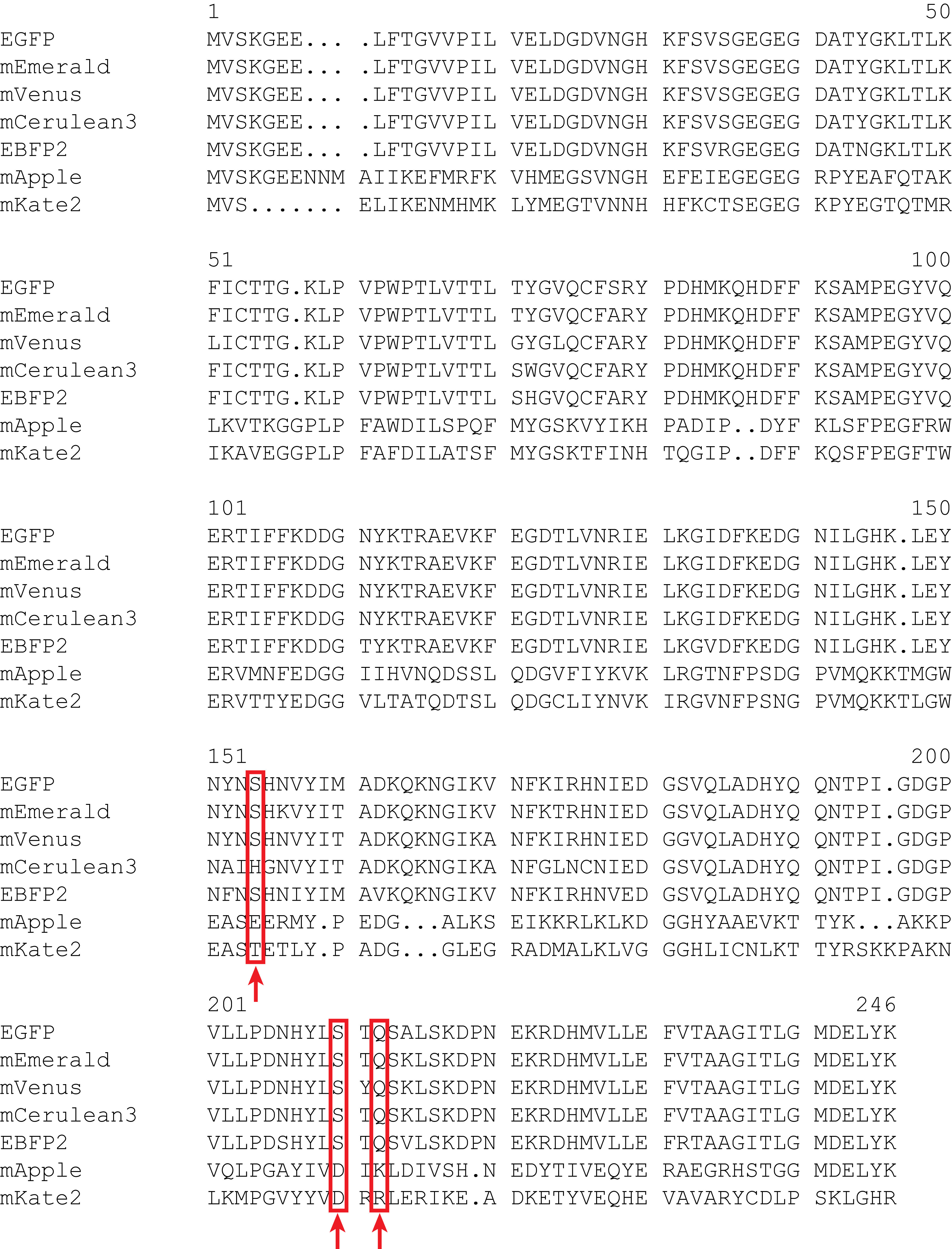

Supplement: Figure S1 — Sequence alignment of all the FPs used in this study, including mEmerald, mVenus, mCerulean3, EBFP2, mApple, and mKate2. The sequence of EGFP is also included as a reference. Red boxes and arrows indicate the mutation sites for the tri-histidine metal binding motif. The numbers are arbitrary sequence numbers that are based on the alignment profile. (TIF) [file pone.0095808.s001.tif]

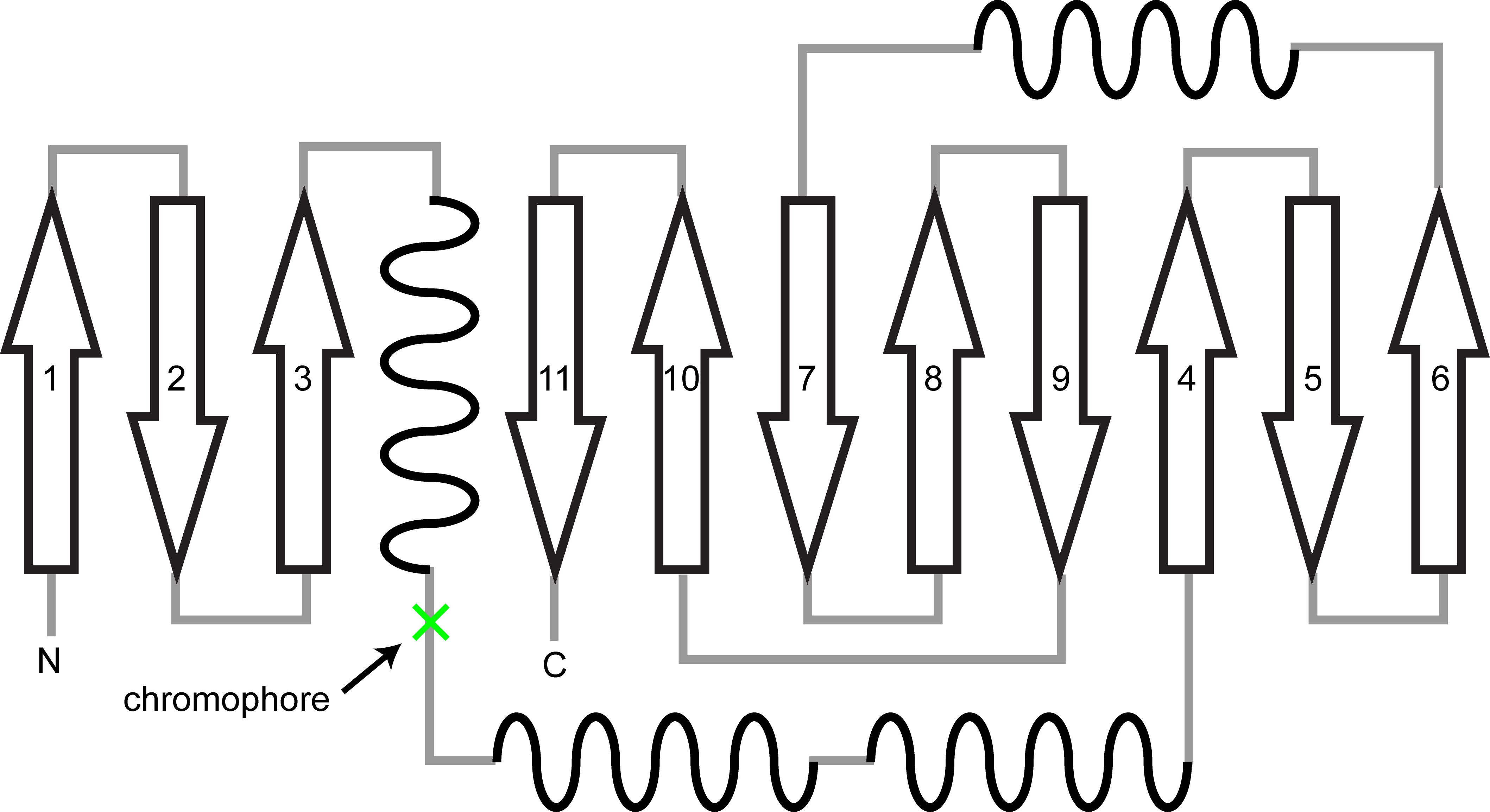

Supplement: Figure S2 — A topology diagram of the iq-mEmerald folding pattern. The β-sheet strands are shown in arrows, α-helices in ribbons, and loops in gray lines. The position of the chromophore is indicated in green and engineered the metal binding histidines are highlighted in red. (TIF) [file pone.0095808.s002.tif]

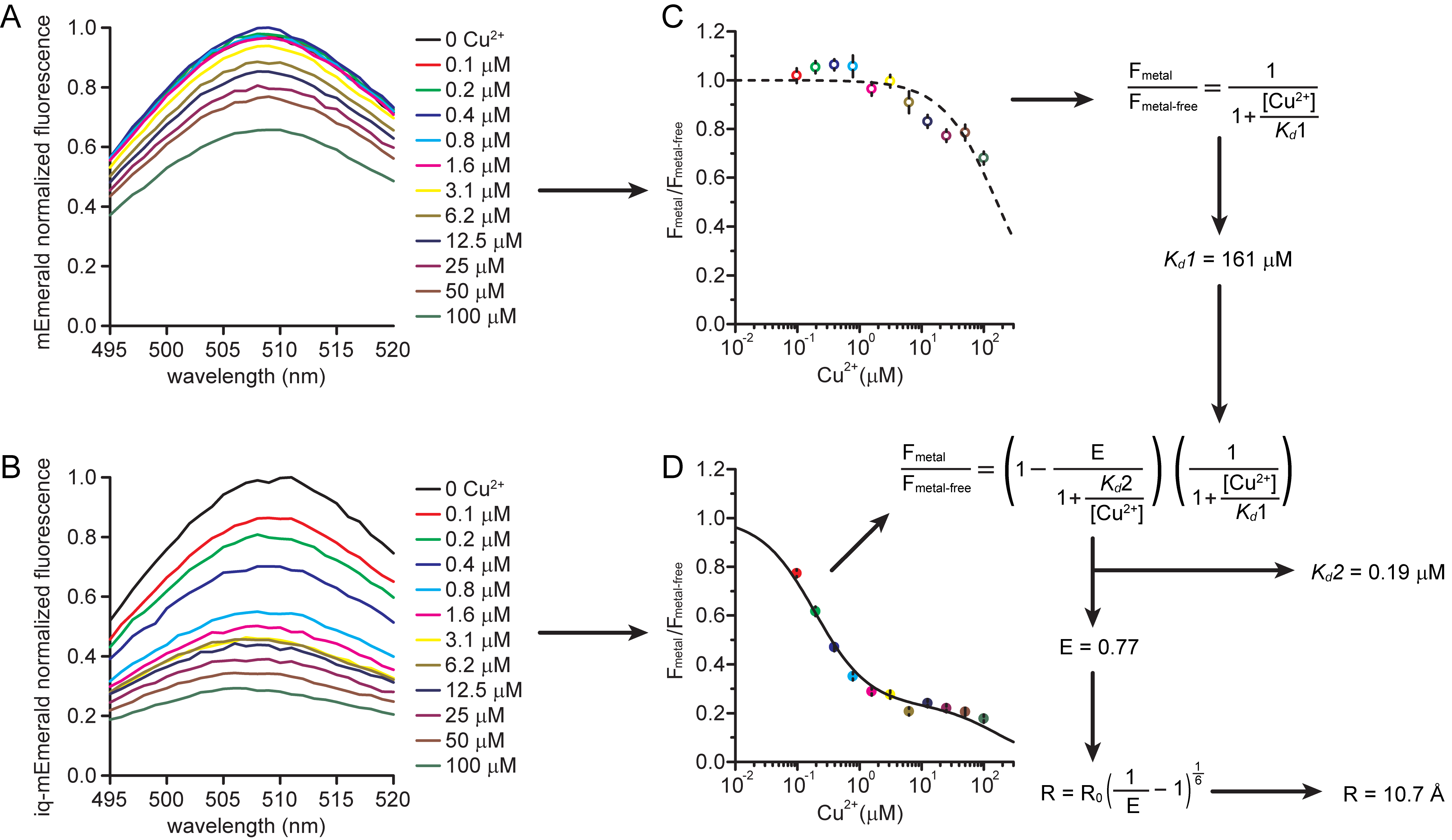

Supplement: Figure S3 — Example data and fit for fluorescence measurements along with the processes to calculate distances. A and B are the fluorescence emission spectra of mEmerald and iq-mEmerald, respectively. The peak intensities are plotted as respect to copper concentrations into C and D. Then the mEmerald quenching data is fitted with a non-specific single binding site equation (shown as the dotted line in C). The fitting result, Kd1 of 161 µM, represents the non-specific quenching of the chromophore by metal solution. Then the quenching of iq-mEmerald is fitted with a two binding site equation (shown as the solid line in D). A binding affinity of 0.19 µM and FRET efficiency of 0.77 are obtained from the fitting. Lastly, the distance (10.7 Å) between the chromophore of iq-Emerald and the metal ion is calculated using the Förster equation and reported in Table S1. (TIF) [file pone.0095808.s003.tif]

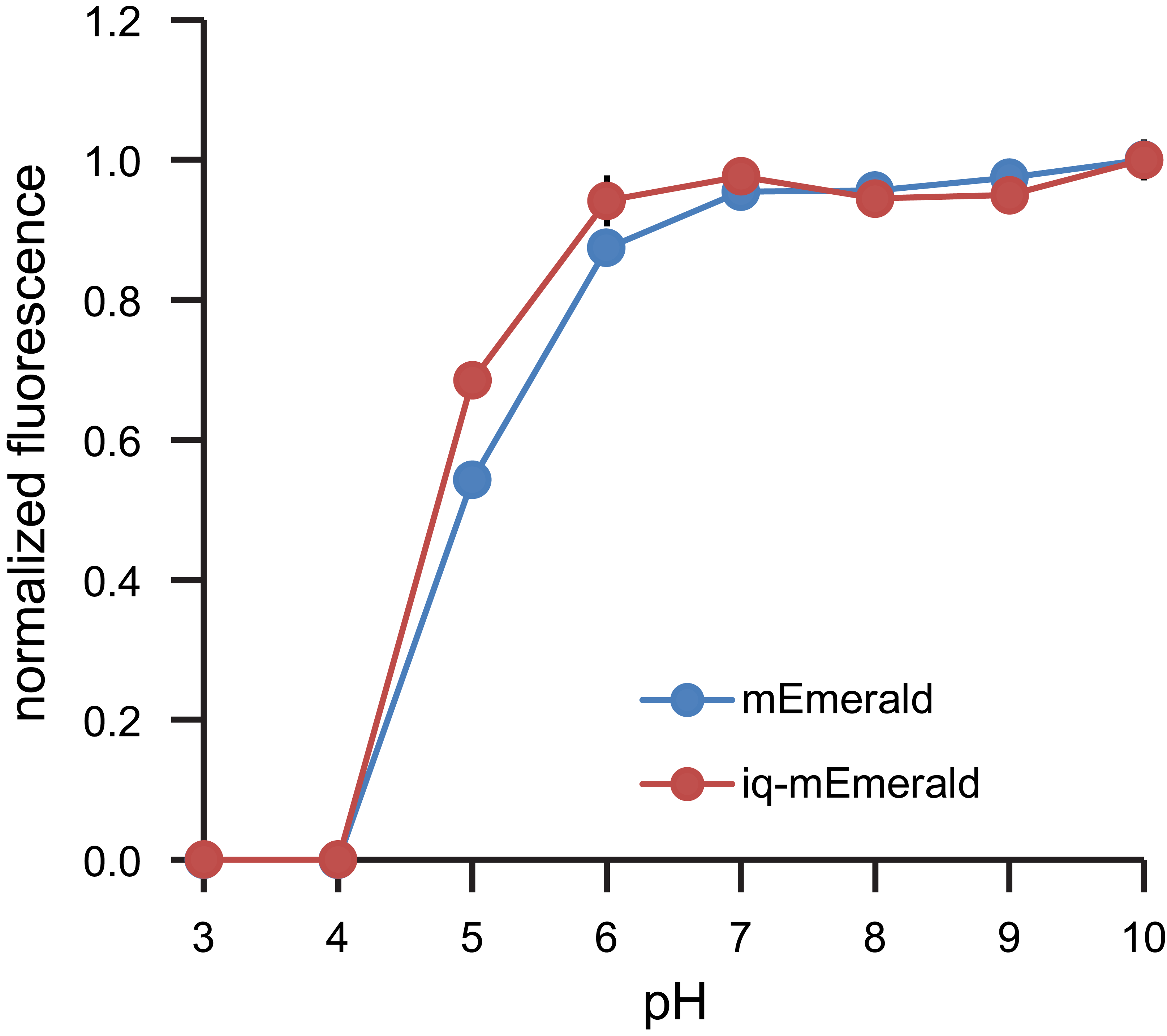

Supplement: Figure S4 — Comparison of the pH dependence of mEmerald (blue) and iq-mEmerald (red). 500 nM of each protein was incubated with different pH buffer solutions. No difference in the pH dependence of fluorescence was observed in iq-mEmerald. (TIF) [file pone.0095808.s004.tif]

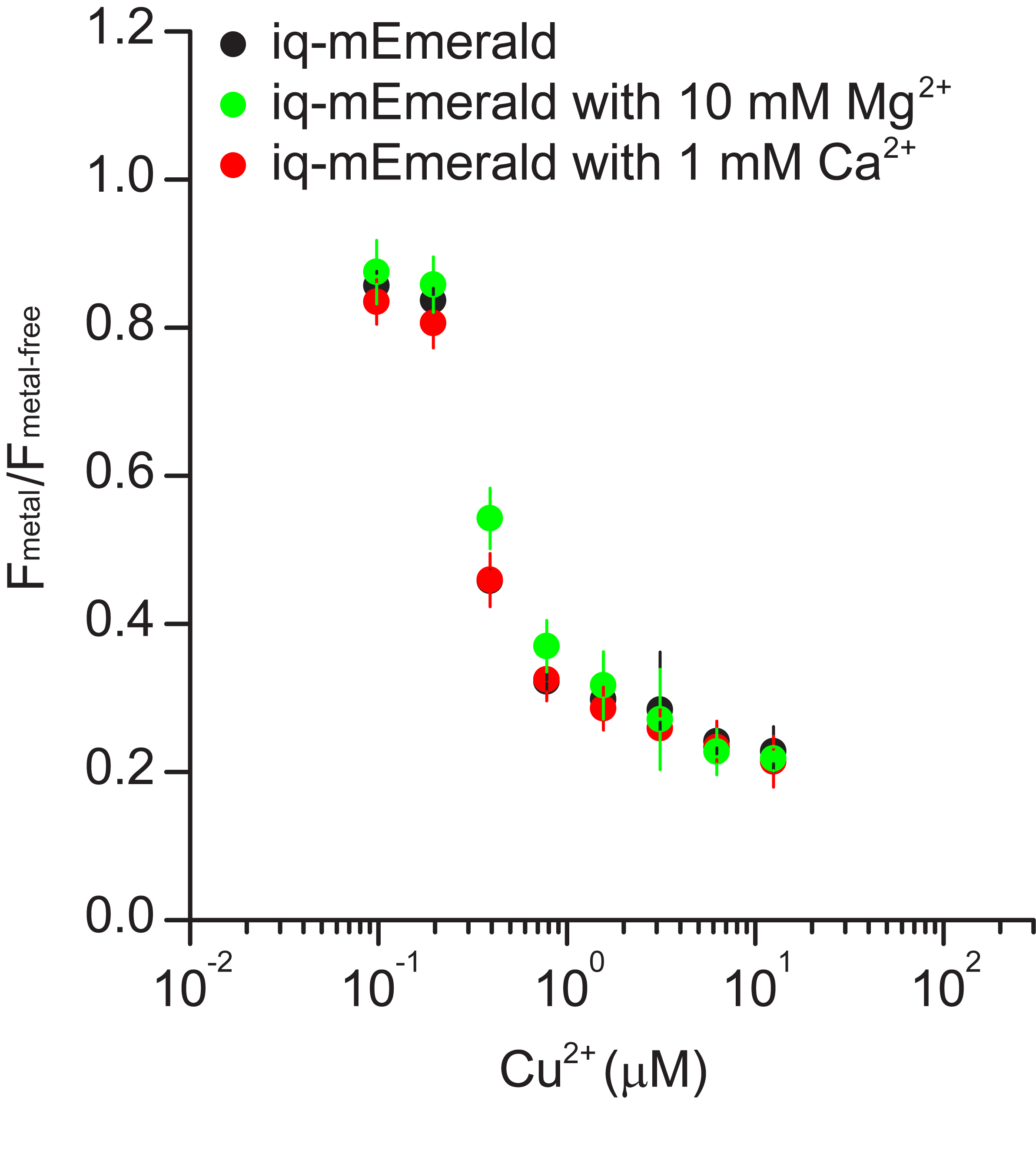

Supplement: Figure S5 — Quenching curves for iq-mEmerald by copper ions without (black) or with the presence of physiological concentrations of calcium (1 mM, red) and magnesium (10 mM, green) ions. Spectra are normalized to the fluorescence without metal and the relative fluorescence from each FP is plotted as a function of copper concentration. (TIF) [file pone.0095808.s005.tif]

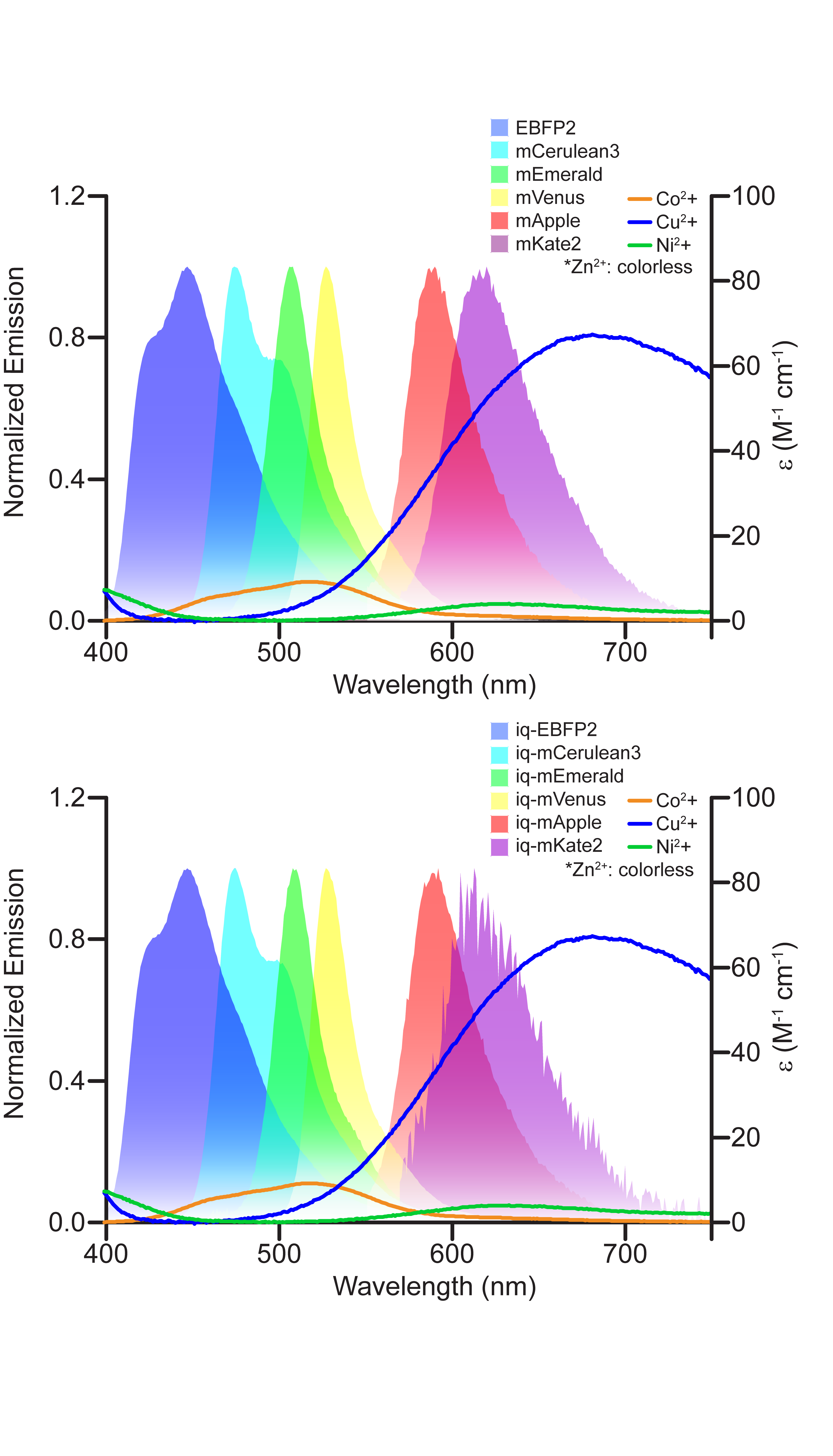

Supplement: Figure S6 — Comparison between the emission spectra of FPs (top) and iq-FPs (bottom) used in this study. The spectra are nearly identical. The absorbance spectra of three color transition metal ions, Co2+, Cu2+, and Ni2+ are plotted for reference. (TIF) [file pone.0095808.s006.tif]

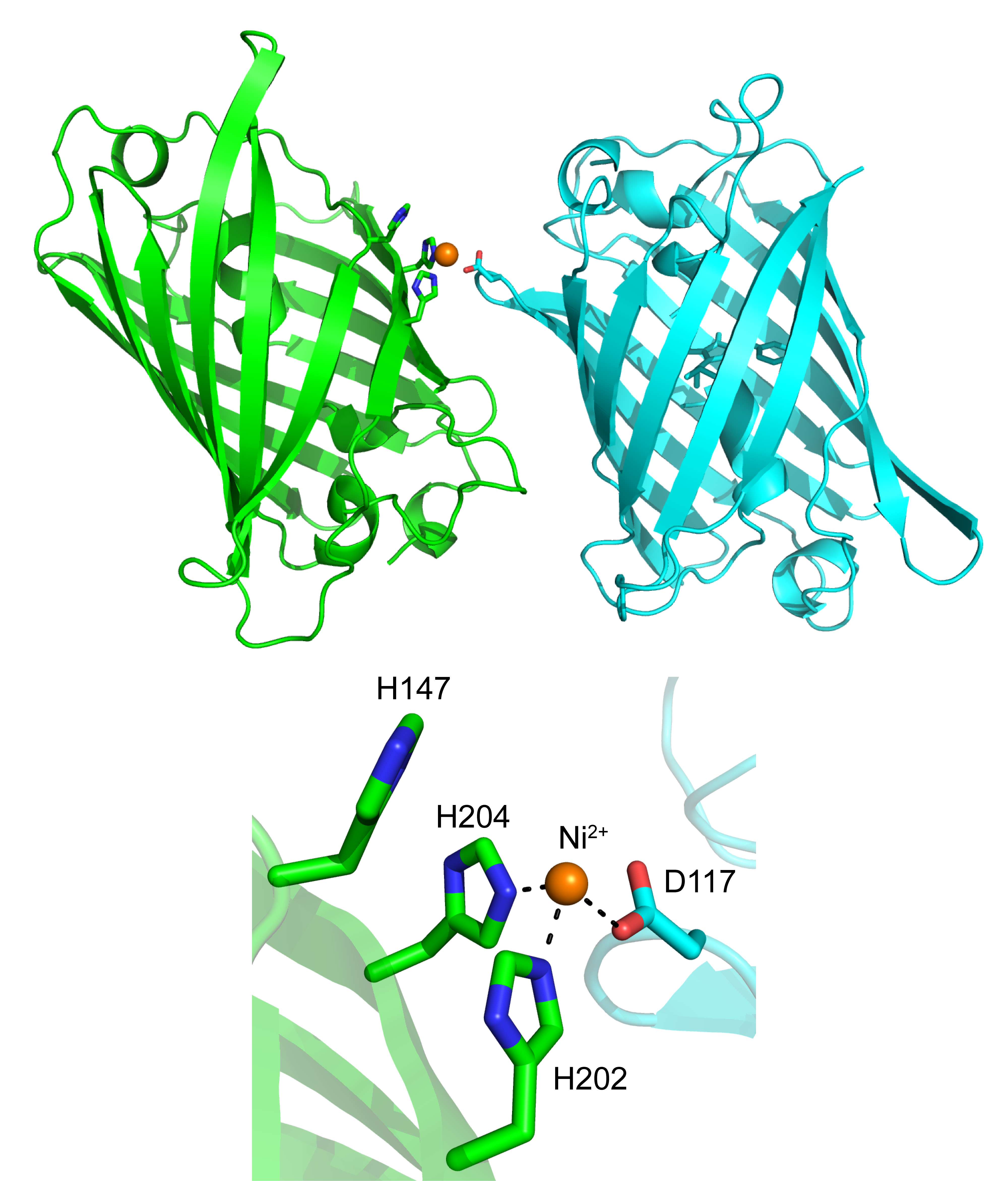

Supplement: Figure S7 — (Top) Crystal contact interactions between two adjacent iq-mEmerald molecules. (Bottom) Zoom-in of the nickel-bound crystal structure. H202 and H204, along with D117 from the neighboring molecule, made direct connect with the nickel ion. H147 was not able to bind this nickel atom due to this crystal contact. (TIF) [file pone.0095808.s007.tif]

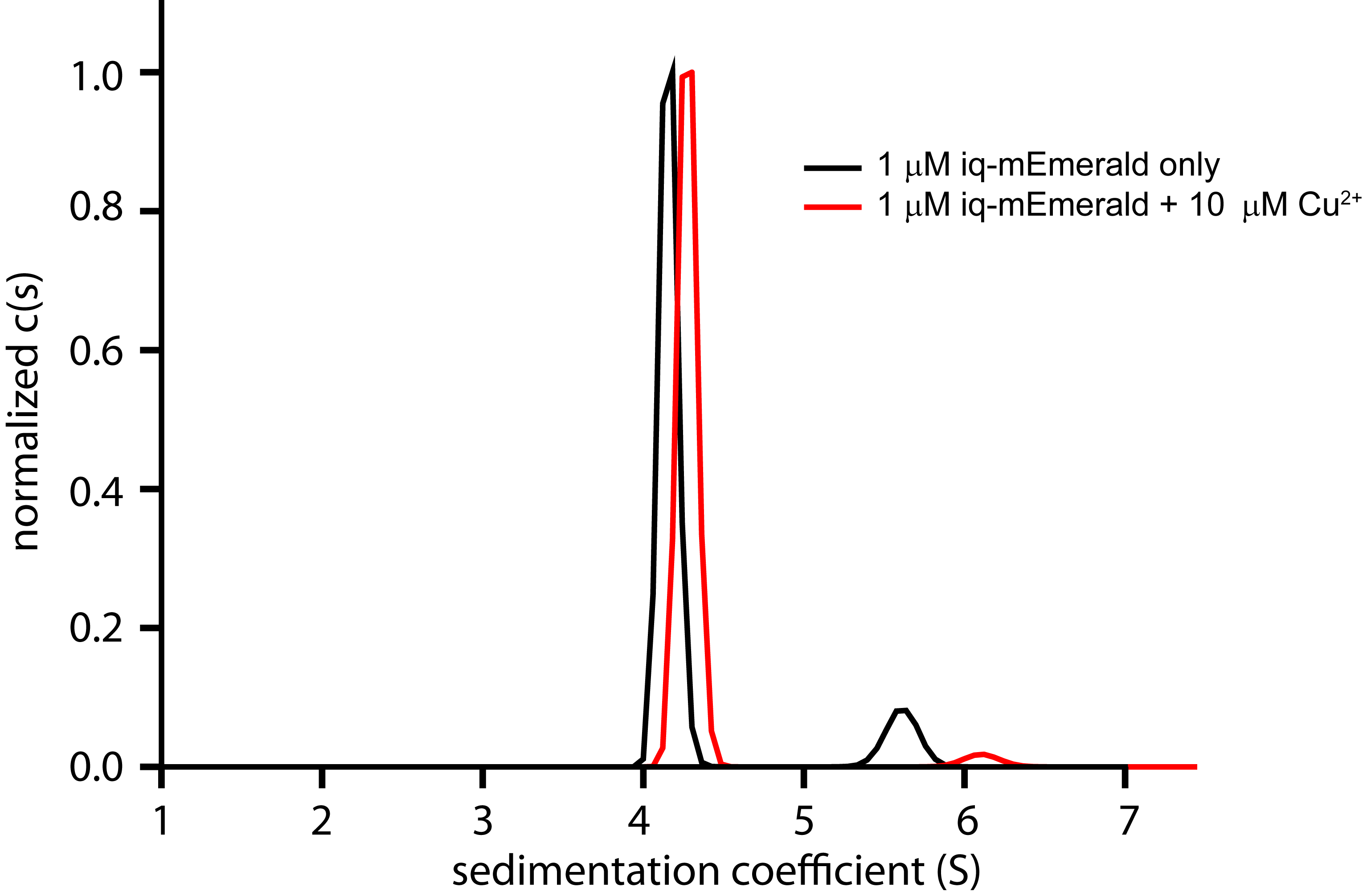

Supplement: Figure S8 — Overlay of the sedimentation coefficient distributions for 1 µM iq-mEmerald (black) and 1 µM iq-mEmerald with 10 µM Cu2+ (red). The mass average s values for the monomer peaks in the Emerald c(s) are 4.15S and 4.27S, for the apo- and Cu-bound iq-mEmerald, respectively. The similar sedimentation coefficient distribution of iq-mEmerald indicates that metal ions do not dimerize iq-mEmerald. (TIF) [file pone.0095808.s008.tif]

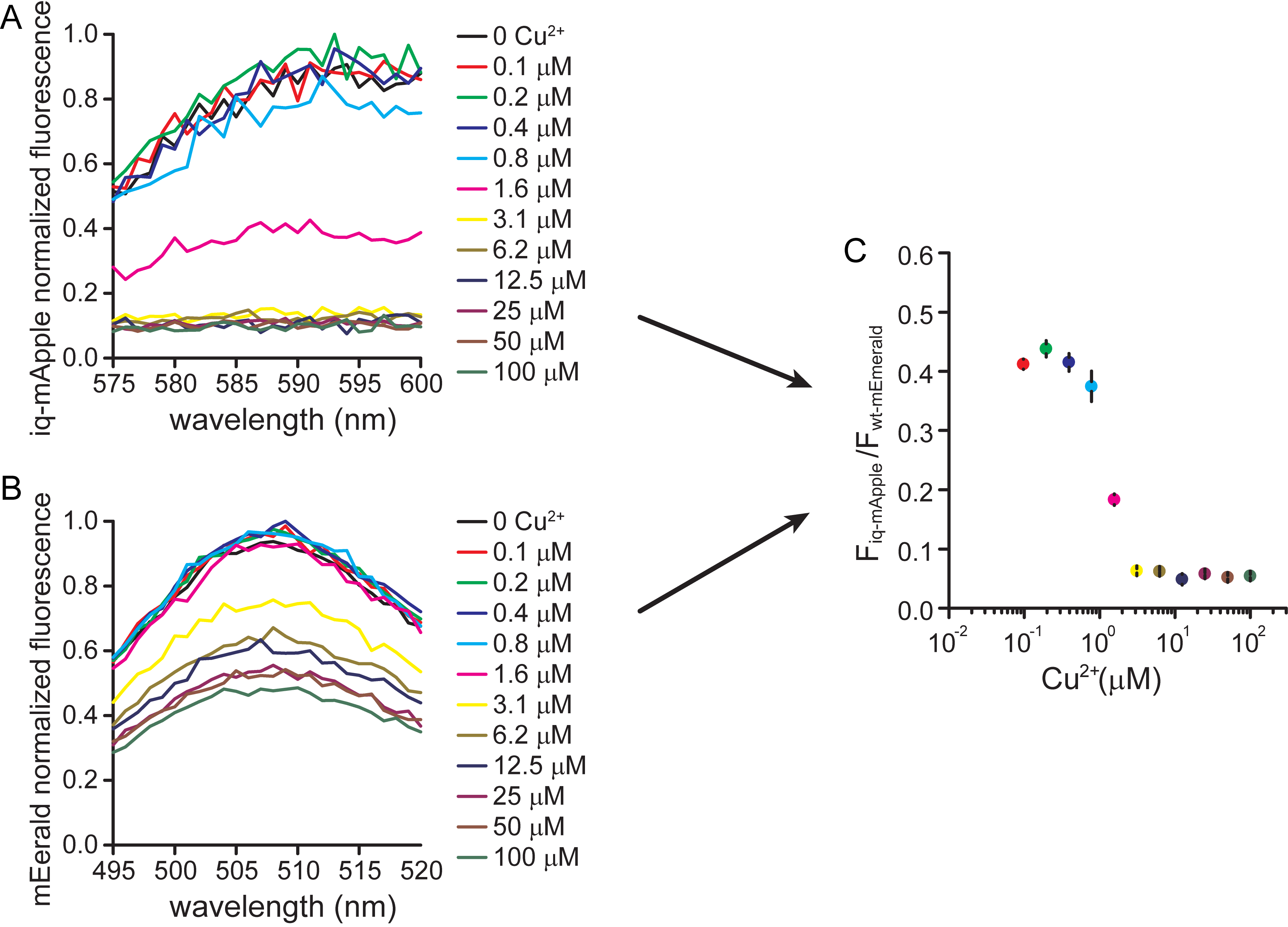

Supplement: Figure S9 — Process used to generate the quenching curves in Figure 3 . This example is the measurement of a ratiometric chimera construct, iq-mApple/mEmerald (middle panel on the first row of Figure 3). A and B are the normalized emission spectra from the contribution of iq-mApple and mEmerald of the chimear, respectively. The peak fluorescence of both underdifferent copper concentrations are taken and plotted as a fluorescent ratio of Fiq-mApple/FmEmerald in C and reported in Figure 3. The excitation wavelengthes for this dimeric construct were the same as the single iq-FPs (432 nm for Emerald and 517 nm for mApple in this example). (TIF) [file pone.0095808.s009.tif]

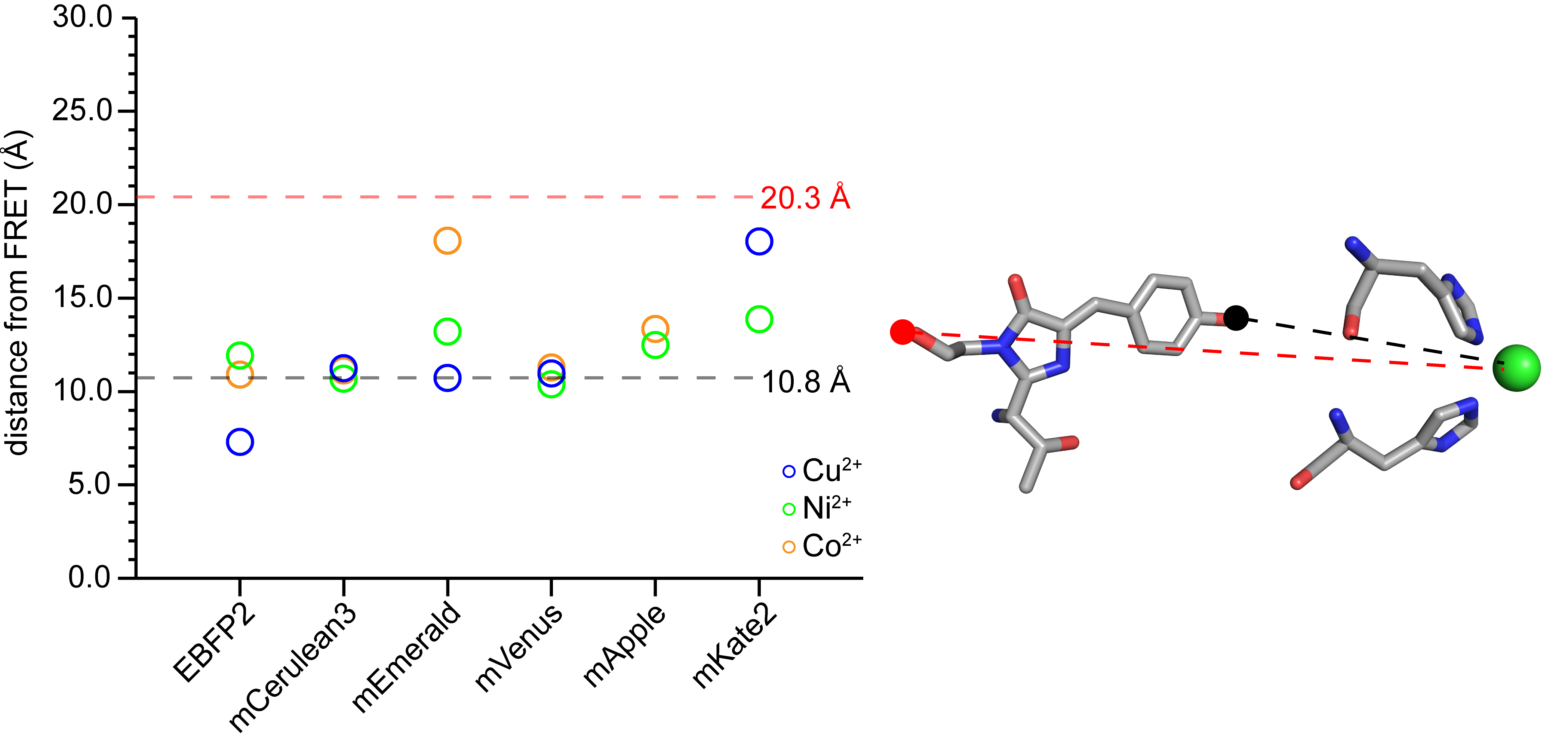

Supplement: Figure S10 — Plot of the distances between FRET donor (chromophores of iq-FPs) and acceptors (metal ions) pairs, calculated from FRET measurements. The dotted lines are the reference distances measured from the nickel-bound iq-mEmerald crystal structure. In this crystal structure, the distance between the nickel ion to the closest and furthest atom on the chromophore are 10.8 and 20.3 Å, respectively. Despite comparing different FP and metal types, most of the FRET calculated distances are within the range that the crystal structure indicated. (TIF) [file pone.0095808.s010.tif]
