# Supplementary material for: An Engineered Palette of Metal Ion Quenchable Fluorescent Proteins
Source: PLoS One. 2014 Apr 21;9(4):e95808. doi: 10.1371/journal.pone.0095808 (PMC3994163; doi:10.1371/journal.pone.0095808)
Supplement: Table S1 — The spectrally calculated R0 and distances calculated from FRET measurements for each iq-FP/metal pair. (PDF) [file pone.0095808.s011.pdf]

Table S1. The spectrally calculated R_0 and distances calculated from FRET measurements for each iq-FP/metal pair.

	iq-FPs/copper		iq-FPs/nickel		iq-FPs/cobalt	
	R_0 (Å)	distance from FRET (Å)	R_0 (Å)	distance from FRET (Å)	R_0 (Å)	distance from FRET (Å)
iq-EBFP2	7.2	7.3	8.5	11.9	11.8	10.9
iq-mCerulean3	12.3	11.2	9.0	10.7	14.1	11.1
iq-mEmerald	13.1	10.7	9.2	13.2	13.9	18.1
iq-mVenus	15.1	11.0	10.3	10.4	13.8	11.3
iq-mApple	18.4	n.a.	12.3	12.5	10.7	13.3
iq-mKate2	20.0	18.0	12.8	13.9	10.1	n.a.
