# Supplementary material for: An Engineered Palette of Metal Ion Quenchable Fluorescent Proteins
Source: PLoS One. 2014 Apr 21;9(4):e95808. doi: 10.1371/journal.pone.0095808 (PMC3994163; doi:10.1371/journal.pone.0095808)
Supplement: Table S2 — Data collection and refinement statistics of the iq-mEmerald crystal structures. (PDF) [file pone.0095808.s012.pdf]

Table S2. Data collection and refinement statistics of the iq-mEmerald crystal structures

	apo iq-mEmerald	iq-mEmerald (Zinc bound)	iq-mEmerald (Nickel bound)
**Data collection**			
Space group	$P\ 4_1\ 2_1\ 2$	$P\ 4_1\ 2_1\ 2$	$P\ 2_1\ 2_1\ 2_1$
Cell dimensions			
a, *b*, *c* (Å)	72.90, 72.90, 113.97	72.80, 72.80, 114.9	51.62, 63.02, 66.35
α, β, γ (°)	90.00, 90.00, 90.00	90.00, 90.00, 90.00	90.00, 90.00, 90.00
Resolution (Å)	1.75 (1.81 - 1.75)	1.8 (1.86 - 1.8)	2.45 (2.54 - 2.45)
R_{sym}	0.088 (0.822)	0.102 (0.765)	0.174 (0.889)
$I / \sigma I$	18.7 (2.63)	12.0 (2.07)	7.55 (1.93)
Completeness (%)	99.9 (99.8)	100.0 (99.9)	99.9 (98.6)
Redundancy	8.3 (7.2)	6.3 (5.5)	6.9 (6.7)
**Refinement**			
Resolution (Å)	1.75	1.8	2.45
No. reflections	31878	29320	8272
R_{work} / R_{free}	0.170 / 0.205	0.176 / 0.211	0.152 / 0.225
No. atoms			
Protein	1850	1717	1679
Ligand/ion	22	28	23
Water	303	214	83
B-factors			
Protein	23.0	23.8	26.9
Ligand/ion	15.7	21.5	24.9
Water	36.4	33.6	27.2
R.m.s deviations			
Bond lengths (Å)	0.011	0.011	0.013
Bond angles (°)	1.40	1.40	1.52

*Data for each structure were obtained from a single crystal
