# Supplementary material for: An Engineered Palette of Metal Ion Quenchable Fluorescent Proteins
Source: PLoS One. 2014 Apr 21;9(4):e95808. doi: 10.1371/journal.pone.0095808 (PMC3994163; doi:10.1371/journal.pone.0095808)
Supplement: Table S3 — The dynamic range and relative fluorescence ratio change (Rmax/Rmin) in three ratiometric metal sensors, iq-mKate2/mCerulean3, iq-mApple/mEmerald, and iq-EBFP2/mEmerald. (PDF) [file pone.0095808.s013.pdf]

Table S3. The dynamic range and relative fluorescence ratio change (R_{max}/R_{min}) in three ratiometric metal sensors, iq-mKate2/mCerulean3, iq-mApple/mEmerald, and iq-EBFP2/mEmerald, are summarized.

metal	iq-mKate2/mCerulean3		iq-mApple/mEmerald		iq-EBFP2/mEmerald	
	dynamic range (μM)	R_{max}/R_{min}	dynamic range (μM)	R_{max}/R_{min}	dynamic range (μM)	R_{max}/R_{min}
Cu^{2+}	0.5 - 10	3.3	0.5 - 5	6.6	n.a.	n.a.
Co^{2+}	5 - 1000	1.3	n.a.	n.a.	0.2 - 200	2.1
Ni^{2+}	2 - 1000	1.5	1 - 100	1.8	0.05 - 2	1.2
Zn^{2+}	0.1 - 5	1.5	n.a.	n.a.	0.01 - 1	1.2
